# Weaker semantic language lateralization associated with better semantic language performance in healthy right‐handed children

**DOI:** 10.1002/brb3.1072

**Published:** 2018-10-08

**Authors:** Lisa Bartha‐Doering, Kathrin Kollndorfer, Gregor Kasprian, Astrid Novak, Anna‐Lisa Schuler, Florian Ph. S. Fischmeister, Johanna Alexopoulos, William Davis Gaillard, Daniela Prayer, Rainer Seidl, Madison M. Berl

**Affiliations:** ^1^ Department of Pediatrics and Adolescent Medicine Medical University of Vienna Vienna Austria; ^2^ Department of Biomedical Imaging and Image‐guided Therapy Medical University of Vienna Vienna Austria; ^3^ Institute of Psychology University of Graz Graz Austria; ^4^ Department of Psychoanalysis and Psychotherapy Medical University of Vienna Vienna Austria; ^5^ Center for Neuroscience and Behavioral Health Children's National Health System (CNHS) Washington DC USA

**Keywords:** functional neuroimaging, language, lateralization, memory

## Abstract

**Introduction:**

The relationship between language abilities and language lateralization in the developing brain is important for our understanding of the neural architecture of language development.

**Methods:**

We investigated 35 right‐handed children and adolescents aged 7–16 years with a functional magnetic resonance imaging language paradigm and a comprehensive language and verbal memory examination.

**Results:**

We found that less lateralized language was significantly correlated with better language performance across areas of the brain and across different language tasks. Less lateralized language in the overall brain was associated with better in‐scanner task accuracy on a semantic language decision task and out‐of‐scanner vocabulary and verbal fluency. Specifically, less lateralized frontal lobe language dominance was associated with better in‐scanner task accuracy and out‐of‐scanner verbal fluency. Furthermore, less lateralized parietal language was associated with better out‐of‐scanner verbal memory across learning, short‐ and long‐delay trials. In contrast, we did not find any relationship between temporal lobe language laterality and verbal performance.

**Conclusions:**

This study suggests that semantic language performance is better with some involvement of the nondominant hemisphere.

## 1 INTRODUCTION

Since the first postmortem lesion studies of patients with aphasia, we know that the left hemisphere carries many language functions (Broca, 1861; Wernicke, 1874). Modern neuroimaging methods demonstrate that in about 90%–96% of adult right‐handers, language is localized predominantly in the left hemisphere (Berl, Zimmaro, et al., 2014; Cabeza & Nyberg, 2000; Greve et al., 2013; Springer et al., 1999). In addition, many left‐handers also show left language dominance (Bartha et al., 2003; Szaflarski et al., 2002). Language lateralization starts very early during neural development. It can already be found in neonates (Peña et al., 2003; Sato et al., 2012; Vannasing et al., 2016) and 3‐month‐old infants (Dehaene‐Lambertz, Dehaene, & Hertz‐Pannier, 2002) and gradually increases with age (Holland et al., 2007; Sepeta et al., 2016; Szaflarski, Holland, Schmithorst, & Byars, 2006). However, even in right‐handers, the degree of language lateralization shows considerable interindividual differences (Berl, Zimmaro, et al., 2014; Springer et al., 1999).

Hemispheric structural asymmetry is not only found in humans, but also in many other species, including birds, mammals, amphibians, fishes, and even bees (Letzkus et al., 2006; Vallortigara & Rogers, 2005), suggesting a selection advantage for more lateralized brains (Hirnstein, Leask, Rose, & Hausmann, 2010). Based on these findings, it may be hypothesized that more lateralized language processing is associated with better language performance. However, studies investigating this hypothesis provide inconclusive results. Using visual field experiments or functional magnetic resonance imaging (fMRI) in healthy adults, some studies report that language lateralization toward the left hemisphere is associated with better language functioning (Chiarello, Welcome, Halderman, & Leonard, 2009; Mellet et al., 2014). Increased left frontal activations are associated with better verbal encoding (Wagner, Pare‐Blagoev, Clark, & Poldrack, 2001; Wagner et al., 1998) and better reading comprehension (Shankweiler et al., 2008). Another study using dichotic listening found a U‐shaped relationship between the degree of language lateralization and performance; higher accuracy was related to asymmetric language lateralization, irrespective of the hemispheric side (Hirnstein, Hugdahl, & Hausmann, 2014). In contrast to this finding, van Ettinger‐Veenstra et al. (2010) reported higher performance in language tasks with more right‐hemisphere involvement and thus suggested a negative linear relationship between language lateralization and language abilities. Finally, in a large study of healthy adults using functional transcranial Doppler sonography (TCD), language lateralization was not correlated with language performance measures (Knecht et al., 2000).

In healthy children, only a few studies investigating the association of language lateralization and language performance are available, and they too provide mixed findings. Everts et al. (2009) observed a positive correlation between verbal IQ and leftward laterality during a vowel detection fMRI task. Groen, Whitehouse, Badcock, and Bishop (2012) reported a significant positive correlation between left language lateralization (using functional TCD) and stronger vocabulary and better nonword reading in healthy children between 6 and 16 years of age. The studies of Berl and colleagues (Berl et al., 2010; Berl, Mayo, et al., 2014) suggest that the relationship between language lateralization and language performance may be task and region dependent. Using an auditory description decision task during fMRI, Berl, Mayo, et al. (2014) showed that a stronger left‐hemispheric lateralization of the temporal region was modestly associated with better object naming, and increased lateralization to the right cerebellum was significantly correlated with better core language performance. In a second study, Berl et al. (2010) used both reading and listening comprehension fMRI tasks to investigate children aged 7–12. They found that greater left frontal lateralization during reading fMRI was associated with better postscan performance, and frontal activation during story listening was positively correlated with better performance on comprehension questions, whereas temporal activations during both fMRI tasks showed no correlations with post‐task performance or cognitive skills. In contrast, Lidzba, Schwilling, Grodd, Krageloh‐Mann, and Wilke (2011) found a significant negative correlation between verbal IQ and language lateralization in an fMRI language comprehension task and interpreted their findings as higher verbal IQ being associated with more right‐hemispheric neural involvement.

Taken together, there is evidence that lateralization of language abilities in the brain may be associated with language performance in both adults and children. However, the nature of this relationship remains unclear. Differences in imaging techniques, lateralization tasks, and cognitive tasks, as well as the focus on different brain regions, may be reasons for inconsistent findings. In addition, only few studies investigated the association of language lateralization and out‐of‐scanner language abilities in healthy children. Furthermore, the overlap and association of the in‐scanner language task with language measures outside the scanner are often unclear.

Information about the relationship between developing language abilities and their representation in the brain is important for our understanding of the neural architecture of language and language development. In this study, we investigated 35 children and adolescents with an fMRI language paradigm for semantic language localization and with a comprehensive language examination. We were especially interested in the relationship between language lateralization in different lobes with in‐scanner task accuracy and various out‐of‐scanner verbal abilities in children. We hypothesized that a greater language lateralization toward the left hemisphere would be associated with better verbal abilities in in‐ and out‐of‐scanner tasks. More specifically, we assumed a strong relationship between in‐scanner task accuracy and all verbal out‐of‐scanner measurements. Furthermore, we assumed associations of a stronger left frontal language laterality associated with better verbal fluency, a stronger left temporal language laterality with better vocabulary, and a stronger overall language laterality with verbal memory.

## 2 METHODS

### 2.1 Participants

Thirty‐five healthy children and adolescents were recruited from community through flyers (for demographic information, see Table 1). All participants met the following criteria: native German speaking, normal hearing, normal or corrected‐to‐normal vision, no history of neurological disease, and no clinical evidence of neurological dysfunction or developmental delay. All participants were right‐handed, measured with the Edinburgh Handedness Inventory EHI (Oldfield, 1971). Participants had a normal Perceptual Reasoning Index with a mean index of 106.46 (range 81–125, *SD* 10.77), comprising three subtests: Block Design, Matrix Reasoning, and Picture Completion of the Hamburg‐Wechsler Intelligenztest für Kinder HAWIK IV (Petermann & Petermann, 2008). Children received a 30 € voucher for a bookstore. The study was approved by the Ethics Committee of the Medical University Vienna and in accordance with the Helsinki Declaration of 1975. For children, age‐appropriate assent forms were provided, and parents received a parental permission form. All children and parents gave written, informed consent prior to inclusion.

**Table 1:** Demographic information of the sample

*n*	35
Sex (f/m)	14/21
Age mean, *SD* (range)	10.57, 2.49 (7–16)
Handedness mean, *SD* (range)	95.43, 10.67 (+50 to +100)

### 2.2 Data acquisition

#### 2.2.1 fMRI paradigm

During fMRI assessment, the German version of an auditory description definition task was administered. This paradigm has shown reliable language lateralization in healthy children (Berl, Mayo, et al., 2014; Pelletier et al., 2011; Sepeta et al., 2016). In auditory description definition condition, the participants hear the definition of an object followed by a noun and are instructed to press a button each time the definition truly described the noun. For instance, “A long yellow fruit is a banana” (true response) or “Something you sit on is a spaghetti” (not true). This paradigm was designed to elicit comprehension of a phrase, semantic recall, and semantic decision. The control condition consisted of reverse speech, with some items additionally containing a pure tone at the end. The participants were instructed to press the button each time he/she heard the tone. The control condition was designed to control for first‐ and second‐order auditory processing, attention, and motor response. Seventy percent of items were correct targets in both conditions. True and false descriptions were pseudorandomly distributed. Performance in the scanner was monitored by the button‐press. Task accuracy was evaluated by the overall accuracy in the language task and the control task separately.

Three different, age‐adjusted versions of the fMRI paradigm were available (7–9 years old, 10–12 years old, 13–16 years old). The difficulty levels were achieved by manipulating linguistic criteria, including word frequency, word length, and word complexity according to normative word data (http://www.wortschatz-unileipzig.de). We used a block design composed of five language task blocks alternating with five control task blocks. Each block lasted for 40 s and consisted of 10 sentences presented every 4 s. Total fMRI scan time was 6 min 40 s.

#### 2.2.2 MRI image acquisition

All participants were scanned on a 3T Siemens TIM Trio whole‐body MR‐Tomograph combined with the manufacturer's default 12‐channel RF head coil (Siemens Medical Solutions, Erlangen Germany) and equipped with a high‐performance gradient system to support fast, high‐resolution whole‐brain echo‐planar imaging. 3D structural MRI scans were performed using an isocubic magnetization‐prepared rapid gradient‐echo (MPRAGE, T1‐weighted, TE/TR _ 4.21/2300 ms, inversion time 900, with a matrix size of 240 × 256 × 160, voxel size 1 × 1 × 1.10 mm, flip angle 9°) sequence. FMRI was acquired using a phase‐corrected blipped gradient‐echo, single‐shot echo‐planar imaging (EPI) sequence. Altogether, 200 EPI volumes were acquired with a square FOV of 210 mm, voxel size 2.1 × 2.1 × 4 mm, 25 percent gap, and 20 slices aligned parallel to the AC‐PC plane using a repetition time (TR) of 2,000 ms, echo time (TE) 42 ms, and a flip angle of 90°.

#### 2.2.3 Out‐of‐scanner cognitive examinations

Verbal abilities were assessed using standardized tests of vocabulary, verbal memory, and verbal fluency. Tests were chosen which examine functions important for language consolidation and vocabulary growth (Deák, 2014), and which are sensitive enough to depict subtle variations in normal cognitive functioning (Thornton & Lukas, 2012). Expressive vocabulary was examined using the Wortschatz‐ und Wortfindungstest WWT (Glück, 2011). This test provides information about expressive vocabulary in different lexical categories including nouns, verbs, and adverbs/adjectives. Immediate verbal auditory attention, short‐term, and working memory were investigated by the digit‐span forward and backward tasks of the HAWIK IV (Petermann & Petermann, 2008). Verbal learning was assessed with the German version of the Auditory Verbal Learning Test (Lezak, 1995), the Verbaler Lern‐ und Merkfähigkeitstest (Helmstädter, Lendt, & Lux, 2001). This test measures the learning efficiency of a list of words, short‐term recall after distraction, long‐term recall, and recognition. Verbal fluency was evaluated using the Regensburger Wortflüssigkeitstest (RWT) (Aschenbrenner, Tucha, & Lange, 2001) which requires the participant to name, within 2 min, as many words as possible of the semantic category animals.

Overall, seven test scores of different cognitive functions were obtained. Raw scores of cognitive tests were converted into *z*‐scores adjusted for age according to the norms of each test. For the WWT vocabulary, norms were only available from 5 to 6–10 to 11 years of age. We therefore transformed the WWT raw scores of the adolescents aged 11–16 into *z*‐scores based on the 10‐ to 11‐year‐old children with the risk of an overestimation of WWT results in the elder participants.

### 2.3 Data analysis

#### 2.3.1 Preprocessing

Preprocessing and statistical analyses were carried out using SPM8 (Wellcome Department of Cognitive Neurology, London, UK) implemented in MATLAB (Version 8.3 Mathworks, Inc., Sherborn, MA, USA). EPI volumes were spatially realigned and corrected for movement. Frame‐to‐frame displacement between successive volumes was estimated by calculating the Euclidian distance from the translational parameters obtained from the realignment. Customized prior probability maps and a customized T1 template, matched to age and gender composition of the study group, were created by employing the Template‐O‐Matic (TOM) toolbox (Wilke, Holland, Altaye, & Gaser, 2008). This approach employs the general linear model and is based on pediatric imaging data from the NIH study on healthy brain development (Evans & Brain Development Cooperative, 2006). It statistically isolates the influence of age or gender on brain structure and thus produces high‐quality matched templates for our pediatric study population. Each subject's anatomical image was segmented with the customized priors and the customized T1 template. After coregistration, the derived spatial normalization parameters were used to normalize the functional volumes. Normalized EPI volumes were smoothed using a spatial filter kernel of FWHM = 8 mm. BOLD signal increases pertaining to task‐evoked responses in brain activity were modeled using a general linear model as implemented in SPM. A regressor modeling residual movement‐related variance (translational and rotational movement) was included in the model as a covariate of no interest. Language activation was measured by contrasting the auditory description definition task condition > reversed language control condition.

Lateralization of activations was estimated at the single‐subject level by use of the LI‐toolbox (Wilke & Lidzba, 2007). To avoid the threshold dependency of simple lateralization indices, a bootstrapping approach was employed. With this approach, a multitude of bootstrapping resamples from the original dataset is analyzed at different thresholds, yielding a single, weighted mean laterality index (LI) which is based on the whole of the underlying dataset (Wilke & Schmithorst, 2006). LIs were computed using the LI‐toolbox masks for different regions of interests (ROI): the global gray matter (LI total), the frontal (LI frontal), temporal (LI temporal), and parietal (LI parietal) lobes separately. LI was calculated according to the formula:

$$\text{LI}=\frac{\left(\sum {\text{activation}}_{\mathrm{left}}\right)/\text{mwf}-\sum {\text{activation}}_{\mathrm{right}}}{\left(\sum {\text{activation}}_{\mathrm{left}}\right)/\text{mwf}+\sum {\text{activation}}_{\mathrm{right}}}$$

where “∑activation” is the sum of activated voxels and “mwf” is the mask weighting factor that represents the relation of the volumes of the masks on the left and on the right to rule out influences of different mask sizes (Wilke & Lidzba, 2007). LI was categorized as left‐lateralized if ≥0.20, bilateral if within −0.20 and +0.20, or right if ≤ −0.20.

A group map of language activation was derived using a one‐sample *t* test corrected for multiple comparisons using the family‐wise error (FWE) method thresholded with *p* < 0.05.

#### 2.3.2 Statistical analyses

Statistical analyses of behavioral data were conducted using SPSS Statistics (version 22.0). As laterality indices and behavioral data were not normally distributed, nonparametric testing was conducted whenever analyses included LIs and/or cognitive test results. The strength of the relationship between continuous variables (cognitive test scores, laterality indices, age at examination) was examined using Spearman's rank correlation coefficient, *r*_s_. Partial nonparametric correlation was calculated using the partial Spearman coefficient *r*_s_. Nonparametric Mann–Whitney *U* test was used to examine whether cognitive test scores or laterality indices differed by sex. Significance of correlations was set based on a Bonferroni correction factor to account for nine cognitive tests, that is, *α* = 0.05/9 = 0.006. Note: We did not consider the different LIs as they were not all independent and highly correlated, please see below.

## 3 RESULTS

### 3.1 Out‐of‐scanner cognitive test results

Out‐of‐scanner cognitive testing revealed average verbal abilities. Means, standard deviations (*SD*), and ranges of age‐adjusted *z*‐scores are depicted in Table 2. There were some children who performed 1 *SD* above and some who performed 1 *SD* below average on a single test; however, it is important to underline that no child showed 1 *SD* below average abilities in more than one area. Overall, these results point to a wide distribution of cognitive performances in our healthy study group.

**Table 2:** Cognitive test results and their correlation with age and strength of handedness, and difference by gender

Cognitive tests	Mean (*SD*)	Range	Correlation with age *r*_s_ (*p*)	Correlation with strength of handedness *r*_s_ (*p*)	Difference by gender *p*
Out‐of‐scanner cognitive tests (*n* = 35)					
Expressive vocabulary	0.49 (1.10)	−2.33 to 2.05	0.28 (0.104)	0.15 (0.401)	0.583
Verbal span	0.19 (0.63)	−0.99 to 1.34	−0.03 (0.876)	−0.31 (0.043)	0.516
Verbal learning efficiency	−0.26 (1.16)	−1.88 to 1.64	0.27 (0.117)	−0.10 (0.585)	0.052
Verbal short‐term memory	0.03 (1.18)	−1.88 to 2.05	0.22 (0.205)	−0.26 (0.131)	0.630
Verbal long‐term memory	0.17 (1.08)	−1.64 to 2.05	0.19 (0.276)	−0.12 (0.487)	0.293
Verbal recognition	−0.32 (0.97)	−1.88 to 1.17	0.53 (0.001)*	−0.13 (0.453)	0.561
Verbal fluency	−0.08 (0.99)	−2.33 to 2.05	0.43 (0.009)	−0.12 (0.499)	0.606
In‐scanner language task (*n* = 24)					
In‐scanner language task performance in percent correct (*n* = 24)	93.83 (7.53)	80 to 100	0.45 (0.026)	−0.12 (0.576)	0.950
In‐scanner reaction time for language task in ms (n = 24)	3,015 (299)	2,339 to 3,739	−0.01 (0.966)	−0.47 (0.020)	0.682

Note

*z*‐Scores of cognitive tests of 35 participants are presented; in‐scanner performance is only available from 24 study participants. Uncorrected *p*‐values are given, and statistical significance after Bonferroni correction is indicated in bold and with *.

Nonparametric correlation analysis revealed a moderate negative relationship between strength of handedness and verbal span (*r*_s_ = −0.31), respectively, but significance did not survive Bonferroni correction. No further correlations were found between strength of handedness and out‐of‐scanner cognitive test results. Age was significantly positively correlated with verbal recognition (*r*_s_ = 0.53, *p* = 0.001), although performance was already age‐corrected. Thus, compared to their specific age groups, older participants in our study were better in verbal recognition than younger ones. In addition, an association between verbal fluency and age was observed (*r*_s_ = 0.43), but significance did not survive Bonferroni correction. There was no other significant correlation between age and verbal performance. Furthermore, verbal performance did not differ by gender.

Correlation between out‐of‐scanner cognitive test results revealed significant correlations among all verbal memory measures (*r*_s_ = 0.53 to 0.83, *p* < 0.001), and between expressive vocabulary and verbal learning (*r*_s_ = 0.53, *p* = 0.001), verbal short‐term memory (*r*_s_ = 0.51, *p* = 0.002), and verbal long‐term memory (*r*_s_ = 0.51, *p* = 0.002), respectively. No significant association surviving Bonferroni correction was found between verbal fluency and any other cognitive measure.

### 3.2 In‐scanner task accuracy

Due to technical reasons, task accuracy for the in‐scanner performance was missing in 11 participants. Mean correct response of 24 participants was 94% (*SD* 7.53) for the auditory description definition condition and 95% (*SD* 6.61) for the control condition. Thus, there was a substantial ceiling effect with regard to in‐scanner task accuracy. Mean reaction time of 24 participants was 3016 ms (*SD* 299) for the auditory description definition condition and 3067 ms (*SD* 245) for the control condition. In‐scanner language task accuracy and reaction time for the language task did not correlate significantly with age indicating proper matching of task demands to age (for a detailed description of demographic and in‐scanner data by age groups, please see the Supporting Information Appendix S1). Furthermore, task performance and reaction time did not differ by gender. There was an association between reaction time and handedness, but correlation did not survive Bonferroni correction (Table 2).

### 3.3 Relationship between in‐scanner performance and out‐of‐scanner cognitive test results

A nonparametric correlation between in‐scanner language task accuracy and reaction time, respectively, and language performance outside the scanner revealed significant positive correlations between the auditory description definition task accuracy in the scanner and the expressive vocabulary (*r*_s_ = 0.58, *p* = 0.003) and verbal fluency abilities tested outside the scanner (*r*_s_ = 0.58, *p* = 0.003). There was no significant association between in‐scanner task accuracy and any out‐of‐scanner verbal memory function. Furthermore, there was no correlation between reaction time for in‐scanner language task accuracy and any out‐of‐scanner cognitive test result (Table 3).

**Table 3:** Correlation of in‐scanner task performance and cognitive test results outside the scanner (*n* = 24)

	Expressive vocabulary	Verbal span	Verbal learning curve	Verbal short‐term memory	Verbal long‐term memory	Verbal recognition	Verbal fluency
In‐scanner language task performance	0.58 (0.003)*	0.14 (0.519)	0.33 (0.118)	0.35 (0.094)	0.26 (0.220)	0.11 (0.602)	0.58 (0.003)*
In‐scanner reaction time for language task	−0.22 (298)	0.30 (0.159)	0.21 (0.336)	0.19 (0.367)	0.08 (0.698)	0.00 (0.989)	0.07 (0.743)

Note

Uncorrected *p*‐values are given, statistical significance after Bonferroni correction is indicated in bold and with *.

### 3.4 fMRI language localization

**Figure 1:**
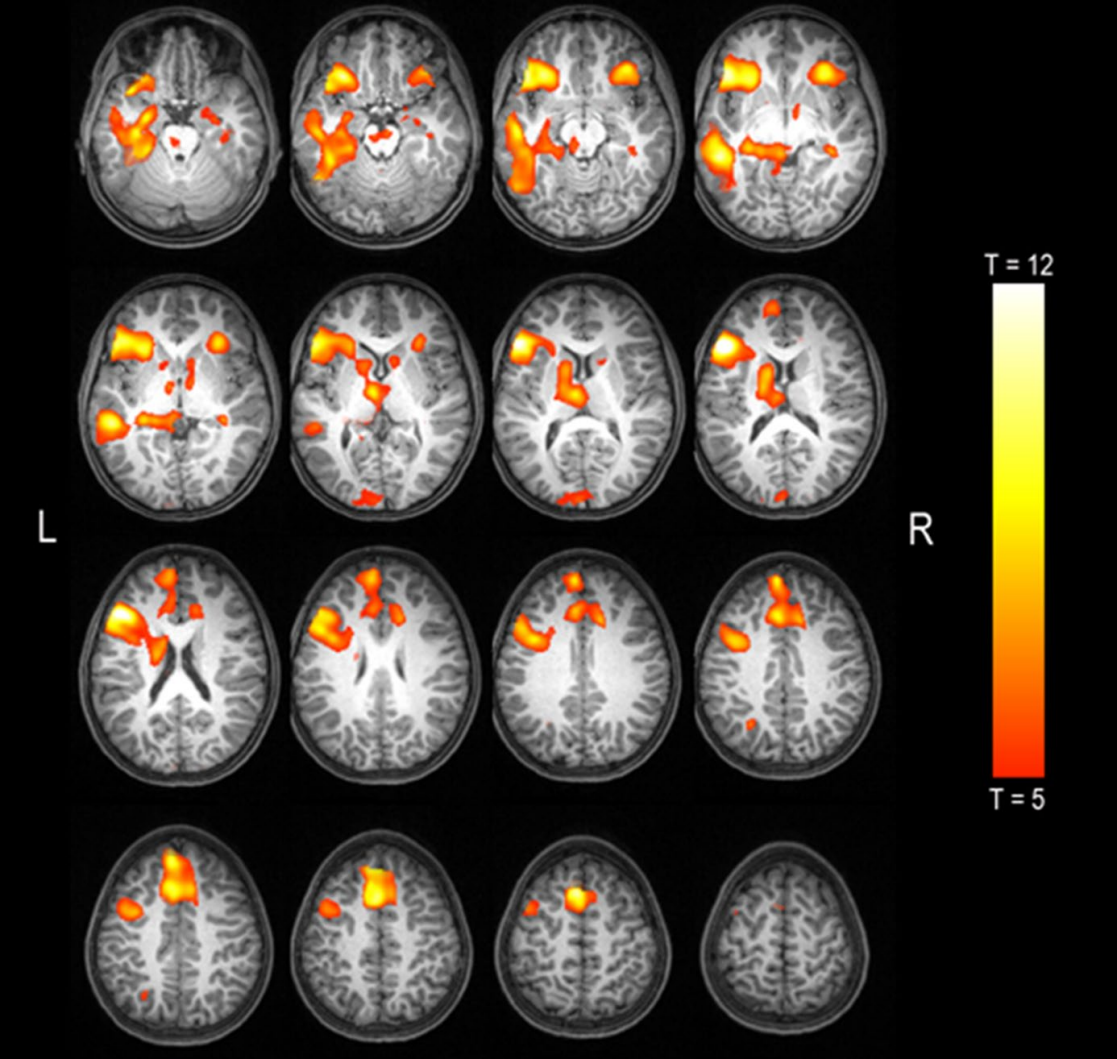
One‐sample *t* test of language activation in the whole group of participants (*p* < 0.05, FWE‐corrected). Group activations are depicted on the normalized T1 of one participant. Left is left hemisphere

Head movement was within the tolerable limit in all children (overall movement group mean 0.07 mm, *SD* 0.04, range 0.03–0.18). In fMRI group analysis, one‐sample *t* test revealed a typical language localization pattern with left‐lateralized activations in lateral and mesial temporal regions including the hippocampus and the parahippocampal gyrus, lateral and mesial frontal regions, and the angular gyrus. In the right hemisphere, the group of participants showed activations in the inferior frontal region and the hippocampal formation (Figure 1 and Table 4).

**Table 4:** Group analysis of language activations

Anatomical region	MNI coordinates			Cluster size	*T*
	*x*	*y*	*z*		
Left hemisphere					
Middle frontal gyrus	−48	26	16	6,338	16.55
Inferior frontal gyrus					
Thalamus					
Middle temporal gyrus	−54	−38	−6	3,917	11.61
Hippocampal gyrus					
Superior frontal gyrus	−4	18	54	2,967	11.19
Medial frontal gyrus					
Angular gyrus	−28	−64	38	40	6.69
Cuneus	−4	−100	8	81	6.37
Isthmus	−16	−48	6	2	5.68
Inferior occipital gyrus	−16	−100	12	2	5.60
Right hemisphere					
Inferior frontal gyrus	32	30	−6	864	9.92
Hippocampus	38	−34	−4	96	8.18
Hippocampus	30	−8	−22	29	6.35
Hippocampus	20	−6	20	1	5.46
Parahippocampal gyrus	38	−22	−22	16	5.81

Note

One‐sample *t* test, FWE‐corrected, *p* < 0.05. Coordinates are given of the peak voxel in activated clusters.

**Figure 2:**
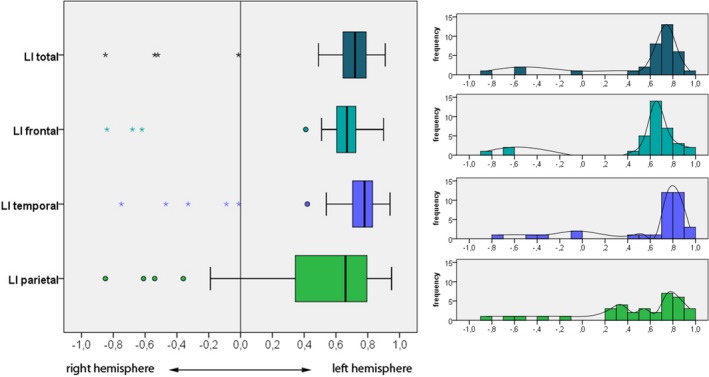
Language laterality indices over the whole group of study participants. Solid vertical lines within bars indicate medians, ° indicates asymmetric outliers, and * indicates extreme outliers (farther than three interquartile ranges)

LIs in single‐subject analyses showed left‐lateralized activations in the overall brain (LI total) in 31 of 35 study participants (Figure 2). This picture was quite the same for the different ROIs: 32 of 35 participants showed a left LI frontal, 30 of 35 a left LI temporal, and 31 of 35 revealed left‐hemisphere language lateralization in the parietal ROI. In contrast, three participants exhibited right‐lateralized activations in LI total, LI frontal, and/or LI temporal, four showed a right‐lateralized LI parietal. One child showed a bilateral LI in LI total, and two participants revealed bilateral LIs in LI temporal. It is important to note that these children with atypical language lateralization in different ROIs were not always the same; overall, six of 35 participants revealed bilateral or right‐lateralized language localization in one or more ROI.

Total LI significantly correlated with frontal LI (*r*_s_ = 0.78, *p* = 0.000), temporal LI (*r*_s_ = 0.61, *p* = 0.000), and parietal LI (*r*_s_ = 0.59, *p* = 0.000), respectively. However, as the total ROI encompasses the other ROIs, it is not independent. Furthermore, we found a significant correlation of frontal LI with parietal LI (*r*_s_ = 0.54, *p* = 0.001), whereas the correlations between frontal LI and temporal LI (*r*_s_ = 0.35, *p* = 0.04) and between temporal LI and parietal LI (*r*_s_ = 0.40, *p* = 0.016) did not survive correction for multiple comparisons.

There were no significant correlations between age and language laterality, neither for LI total nor for the LIs of the different ROIs (Table 5). Furthermore, language laterality did not differ by gender. However, despite the inclusion criterion of right‐handedness in our study sample, a significant correlation of the LI total and the LI temporal, respectively, with the EHI handedness quotient was found (for each *r*_s_ = 0.47, *p* = 0.005). Thus, the stronger the right‐handedness of the participant, the more left lateralized were language activations in the whole cortex and in temporal areas.

**Table 5:** Language laterality indices and their correlation to age and strength of handedness, and difference by gender

Laterality indices	Mean (*SD*)	Median	Range	Age *r*_s_ (*p*)	Strength of handedness *r*_s_ (*p*)	Gender *p*
LI total	0.59 (0.41)	0.72	−0.85 to 0.91	−0.04 (0.808)	0.47 (0.005)*	0.325
LI frontal	0.56 (0.42)	0.67	−0.84 to 0.90	−0.24 (0.164)	0.31 (0.070)	0.678
LI temporal	0.62 (0.42)	0.78	−0.75 to 0.94	0.09 (0.623)	0.47 (0.005)*	0.907
LI parietal	0.48 (0.47)	0.66	−0.85 to 0.95	0.08 (0.642)	−0.01 (0.962)	0.960

Note

Cognitive test scores of 35 participants are presented; in‐scanner performance is only available from 24 study participants. Uncorrected *p*‐values are given. Statistical significance after Bonferroni correction is indicated in bold and with *.

### 3.5 Association of verbal performance with language lateralization

Due to the significant correlation between the strength of handedness and LI total and LI temporal, respectively, as well as the moderate negative association between the strength of handedness and out‐of‐scanner verbal span and in‐scanner reaction time, respectively, we controlled the correlation for strength of handedness. Furthermore, to be conservative, the correlation was also controlled for age, as previous studies found a dependence of LI by age.

**Figure 3:**
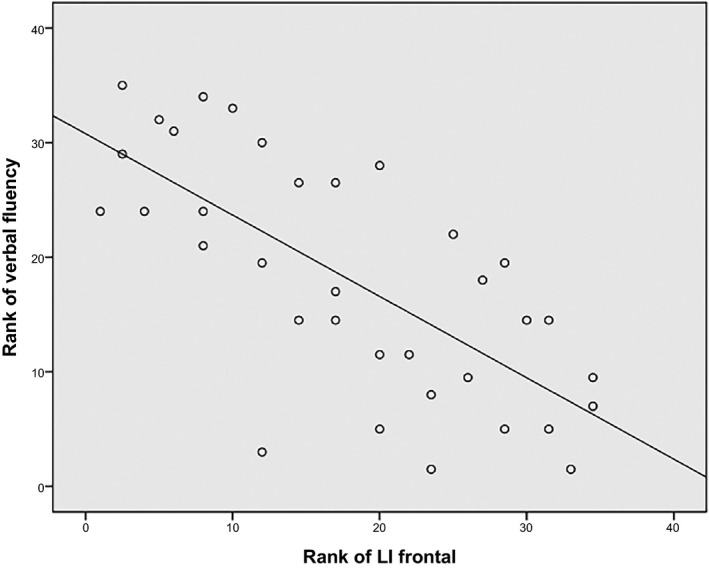
Partial correlation of frontal language laterality index with verbal fluency

Nonparametric correlation analyses revealed strong negative correlations between LI total and out‐of‐scanner vocabulary (*r*_s_ = −0.57, *p* = 0.006) and out‐of‐scanner verbal fluency, respectively (*r*_s_ = −0.63, *p* = 0.002) (Table 6). Furthermore, LI frontal negatively correlated with out‐of‐scanner verbal fluency (*r*_s_ = −0.64, *p* = 0.001) (Figure 3). In addition, LI parietal moderately correlated with the verbal learning curve of the out‐of‐scanner auditory verbal learning test (*r*_s_ = −0.59, *p* = 0.004). Thus, the less left‐lateralized fMRI language activations were, the better out‐of‐scanner performance was. Likewise, we observed a strong negative correlation of in‐scanner task accuracy with LI total (*r*_s_ = −0.81, *p* = 0.000), LI frontal (*r*_s_ = −0.76, *p* = 0.000), and LI parietal (*r*_s_ = −0.62, *p* = 0.002). However, as there was a substantial ceiling effect in the in‐scanner task accuracy, analyses including this variable have to be taken with caution. We did not find any other significant associations between language laterality and verbal performance that survived Bonferroni corrections. Notably, temporal language laterality did not correlate with any measures. Furthermore, we did not find any association between language laterality measures and in‐scanner reaction times.

**Table 6:** Correlation of cognitive test results with laterality indices, controlled for age, and strength of handedness

	LI total *r*_s_ (*p*)	LI frontal *r*_s_ (*p*)	LI temporal *r*_s_ (*p*)	LI parietal *r*_s_ (*p*)
Cognitive tests outside the scanner (*n* = 35)				
Expressive vocabulary	−0.57 (0.006)*	−0.31 (0.162)	−0.45 (0.034)	−0.50 (0.018)
Verbal span	−0.25 (0.258)	0.08 (0.976)	−0.50 (0.018)	−0.20 (0.373)
Verbal learning curve	−0.37 (0.091)	−0.26 (0.242)	−0.32 (0.142)	−0.59 (0.004)*
Verbal short‐term memory	−0.40 (0.067)	−0.14 (0.546)	−0.34 (0.127)	−0.53 (0.011)
Verbal long‐term memory	−0.23 (0.312)	−0.05 (0.842)	−0.21 (0.343)	−0.47 (0.026)
Verbal recognition	−0.03 (0.880)	0.11 (0.617)	−0.15 (0.521)	0.13 (0.560)
Verbal fluency	−0.63 (0.002)*	−0.64 (0.001)*	−0.27 (0.232)	−0.39 (0.076)
In‐scanner language task (*n* = 24)				
Task performance	−0.81 (0.000)*	−0.76 (0.000)*	−0.21 (0.353)	−0.62 (0.002)*
Reaction time	−0.17 (0.452)	−0.17 (0.443)	−0.19 (0.398)	−0.23 (0.296)

Note

Uncorrected *p‐*values are given. Statistical significance after Bonferroni correction is indicated in bold and with *.

## 4 DISCUSSION

While there is a large body of studies investigating language localization in healthy children using fMRI, only a few studies investigated the relationship between language lateralization and out‐of‐scanner language abilities in healthy children and adolescents. The present study was therefore interested in the association of language lateralization and in‐ and out‐of‐scanner language performance in various verbal domains in 35 healthy school‐aged children and adolescents. Similar to previous studies, we found that among right‐handed children and adolescents aged 7–16 years a majority were left‐hemisphere language dominant on a language fMRI task. However, contrary to our study hypothesis, less lateralized semantic language dominance was significantly correlated with better verbal performance in and out of the scanner. Less lateralization in the overall brain was associated with better vocabulary and verbal fluency. Moreover, different regions were correlated with different aspects of verbal performance. Less lateralization of the frontal lobes was associated with better in‐scanner semantic language decision and out‐of‐scanner verbal fluency, and less lateralization of the parietal lobes was associated with better out‐of‐scanner verbal learning. In contrast, temporal areas were not correlated with verbal performance across any measure.

### 4.1 Bilateral language network associated with better language performance

These findings are in concordance with other fMRI studies where better language performance in children was also related to less lateralization and more involvement of the contralateral hemisphere (typically the right homologue) (Lidzba et al., 2011; Yeatman, Ben‐Shachar, Glover, & Feldman, 2010). Perhaps greater interhemispheric communication accounts for better verbal functioning. Due to the relative nature of LI, it is not possible to disentangle between weaker involvement of the dominant hemisphere versus higher involvement of the nondominant hemisphere, with both resulting in more symmetrical LIs (Jansen et al., 2006; Seghier, 2008; Seghier, Kherif, Josse, & Price, 2011; —for a possible alternative, see Wang, Mechanic‐Hamilton, Pluta, Glynn, & Detre, 2009). Thus, the fMRI LI calculation does not rule out the possibility that those children with better cognitive performances have a weaker involvement of left‐hemisphere language regions. Furthermore, larger ROIs tend to lead to less lateralized activation than smaller ROIs as additional cognitive processes might lead to a broader activation during the task. Nevertheless, our primary interpretation of the study results is that children with higher language proficiency use a more bilateral network with increased involvement of the right hemisphere. When accessing a larger semantic knowledge, as is presumably done during the fMRI paradigm, vocabulary task, and verbal fluency, a larger and a more widely distributed functional network of semantic representations may be activated (Lidzba et al., 2011). Studies have revealed that greater interhemispheric connectivity goes along with a larger corpus callosum (Ruddy, Leemans, & Carson, 2017) and that corpus callosum volume is associated with better language and episodic memory functions (Christman & Propper, 2001; Hines, Chiu, McAdams, Bentler, & Lipcamon, 1992). Furthermore, studies investigating the cortical response to increasing task difficulty found an increase in activation volume in bilateral perisylvian regions (Caplan et al., 2002; Just, Carpenter, Keller, Eddy, & Thulborn, 1996; Just & Varma, 2007; Kaan & Swaab, 2002). Remarkably, Yeatman et al. (2010) showed that children with better receptive language skills had a greater increase in both inferior frontal gyri during processing of complex sentences than children with average receptive language skills. The inferior frontal gyrus has been shown to be specifically involved in language tasks with high processing demands (Ben‐Shachar, Hendler, Kahn, Ben‐Bashat, & Grodzinsky, 2003; Ben‐Shachar, Palti, & Grodzinsky, 2004; Fiebach, Schlesewsky, Lohmann, von Cramon, & Friederici, 2005; Friederici, Ruschemeyer, Hahne, & Fiebach, 2003). Thus, children performing at highest levels in language tasks showed more bilateral activation in higher‐order brain areas during a more complex task.

### 4.2 Language lateralization in healthy versus patient populations

Less lateralized language activation may reflect a different underlying mechanism in children with disorders as several studies have found that better linguistic abilities are associated with greater left lateralization of language: Lillywhite et al. (2009) assessed language performance and fMRI patterns of language lateralization in children with benign childhood epilepsy with centrotemporal spikes and found better language performance correlated with increased left‐sided language lateralization. In children with left‐sided focal brain lesions, left‐hemispheric language lateralization was associated with increased language task accuracy (Elkana et al., 2011). In addition, de Guibert et al. (2011) investigated children with developmental dysphasia and found significantly less left lateralization in all core language regions, including the inferior frontal gyrus, supramarginal gyrus, and superior temporal gyrus, compared to healthy controls. Thus, the average degree of lateralization is not similar between healthy control and patient populations. Recruitment of contralateral homologues may therefore represent different underlying phenomenon in healthy or patient populations: In children with language deficits, less lateralization may represent a compensatory strategy, while in children with strong language, less lateralization may represent a superior strategy.

### 4.3 Contradictory findings

There are, nonetheless, some further studies in healthy children reporting a positive relationship between language lateralization toward the left hemisphere and verbal abilities. In an fMRI study, Everts et al. (2009) used two language paradigms, vowel detection, and synonym finding, where especially the latter is supposed to elicit a similar language network compared to our task. They found a significant correlation between the laterality indices of the two fMRI language paradigms and verbal IQ. Besides that the verbal IQ is a gross measure incorporating also arithmetics and digit span, not all study probands in Evert et al.'s study participated in the cognitive testing, yielding data of 15 children and adolescents. It may be questioned whether this correlation holds true in a larger study group (Bhaumik et al., 2009). Groen et al. (2012) used functional TCD and a language production paradigm where children were asked to describe an animation. They found that children with left‐hemisphere language lateralization had better vocabulary and nonword reading skills for their age compared with other children. Furthermore, Berl et al. (2010) found that greater left frontal lateralization during reading comprehension fMRI was associated with better postscan recall questions. The contradictory findings of these studies compared to the results of the present study may be the result of different imaging methods and different tasks testing different functions. The relationship between lateralization and cognitive performance has been reported as dependent on task demands (Boles, Barth, & Merrill, 2008; Piervincenzi et al., 2016).

Task demands may also explain why temporal LI was not associated with verbal performance in our study. The out‐of‐scanner testing included all expressive tasks and did not include much comprehension. Our semantic fMRI task certainly requires temporal activation with comprehension of the sentence; however, it is also ultimately a decision task that relies on frontal activation. Thus, the generalizability of our findings is limited to aspects of language that are engaged by our task. It is likely that fMRI tasks with more phonological emphasis would show temporal lobe correlations, and syntactic tasks may demonstrate more bilateral activations (Binder, Swanson, Hammeke, & Sabsevitz, 2008; Schell, Zaccarella, & Friederici, 2017; Szaflarski et al., 2008).

### 4.4 Language lateralization and age

Theories on the relationship between neural maturation and cognitive development have suggested that while some brain regions become increasingly involved in cognition, the influence of others on cognitive development decreases (Johnson, 2005). Thus, cognition seems to develop in relation to both progressive and regressive neural mechanisms of change. The present study was not able to find a significant association of laterality indices and age. This may be due to the lack of well‐balanced and wide age ranges, but can also be the result of the fact that the in‐scanner cognitive stimulus set was adapted to the age of the participants. Some studies have, however, observed an increase in language lateralization toward the left hemisphere with increasing age (Berl, Mayo, et al., 2014; Brown, Symingtion, VanLancker‐Sidtis, Dietrich, & Paul, 2005; Everts et al., 2009; Lidzba et al., 2011; Szaflarski, Altaye,et al., 2012). It has been hypothesized that this language lateralization increase reflects a specialization of areas which goes along with a gain of proficiency. However, there may be a different explanation for these findings. Children need a high proficiency in language as they have to acquire an enormous amount of phonological, prosodic, syntactic, and verbal information. In absolute measurements, they may perform inferior to adults, but morphosyntax and vocabulary acquisition are much easier for children than for adults, and memorization of unknown verbal material is highly superior in children (Birdsong, 2006; DeKeyser, 2000; Stölten, Abrahamsson, & Hyltenstam, 2014). Children are, in addition, more skilled at identifying subtle differences in sounds and are therefore better in pronunciation learning than adults (Flege & MacKay, 2004). Therefore, we assume that during the time of specialization and consolidation of language abilities where language‐associated areas mature, children recruit a larger neural language network compared to adults. In light of our present findings, we suppose that the larger this network is, the better the function.

### 4.5 Task difficulty and activation increase

Besides in‐scanner task accuracy, we evaluated in‐scanner reaction times during performance. Reaction times may provide an additional measure of task difficulty (Kyllonen & Zu, 2016). Some studies show that right‐hemispheric activations increase when additional resources are needed to sustain tasks with increasing difficulty ([Dima, Jogia, & Frangou, 2014; Postman‐Caucheteux et al., 2010] but see also [Dräger & Knecht, 2002]). However, in the present study, we were not able to identify any associations between in‐scanner reaction times and language lateralization in different regions nor in the overall brain. This suggests that the involvement of the nondominant hemisphere was not due to increased task difficulty in our study participants.

### 4.6 Language lateralization and handedness

Interestingly, our study sample showed a significant positive correlation of language laterality with the strength of handedness. Although all study participants were clearly right‐handed with EHI ranges from +50 to +100, participants with a stronger right‐handedness exhibited more left‐hemisphere language activations. This correlation was significant for the semantic language lateralization evaluated for both the whole brain and the temporal lobes alone. This observation is contrary to the findings of Mazoyer et al. (2014) who investigated the relationship between language lateralization and manual preference in 297 subjects, half of them left‐handed, and found no significant relationship between EHI and language lateralization, except in a small subgroup of strongly atypical lateralizing individuals who were left‐handed. This difference in findings may be a reflection that Mazoyer et al. only calculated language laterality in the overall brain, whereas we analyzed the laterality of language activations for different brain regions. Whereas frontal and parietal LIs were not associated with the strength of manual preference in our study, temporal areas showed a high correlation with the degree of right‐handedness.

As the present study only included right‐handed children and adolescents, its findings cannot be generalized to left‐handed populations. From our data, we hypothesize that less lateralized semantic language dominance is also favorable for verbal performance in left‐handed children and adolescents. On the other hand, previous studies have shown that left‐handed populations are more heterogeneous in both lateralization and performance, and the relationship between functional asymmetry and performance in left‐handers seems to be more complex than in right‐handers (Somers, Shields, Boks, Kahn, & Sommer, 2015; Somers, Aukes, et al., 2015; Szaflarski et al., 2002; Szaflarski, Rajagopal, et al., 2012). Future studies are therefore needed in left‐handed children and ambidextrous to form a comprehensive picture of the relationship between language lateralization and performance in childhood and adolescence.

### 4.7 Limitations

Correlation analyses between language laterality and cognitive measurements were controlled for age effects, and the strong negative correlation between semantic language dominance and different language abilities remained. Nevertheless, the large age range of our study participants presents some challenges. First, the out‐of‐scanner expressive vocabulary test lacks normative data for children older than 11 years. The ability to name the items of this test rapidly increases with age, however, by age ten mean performance is largely flat and at near perfect (Glück, 2011), we thus transformed the raw scores of the elder participants into *z*‐scores based on the 10‐ to 11‐year‐old children. However, for the interpretation of the results concerning the vocabulary test, the risk of an overestimation of *z*‐score results for the elder study participants has to be taken into account. Second, for the in‐scanner fMRI task, three different age‐adjusted versions of the fMRI paradigm were developed. Age adjustment was reached by manipulating linguistic criteria of stimuli, specifically word frequency. As task difficulty is known to modulate brain activity (Dräger et al., 2004), this is a common psycholinguistic method to achieve comparable difficulty levels across age ranges (Ambridge & Rowland, 2013; Cowan, Saults, & Elliott, 2002) and was also used previously in the original, English version of the fMRI task developed by Berl and co‐workers (Berl, Mayo, et al., 2014; Sepeta et al., 2015, 2016; Sun et al., 2013). However, it means that linguistic stimuli differed among age groups. While there was a range of task accuracy within each age group, it was a limited range as task accuracy was quite high. Moreover, task accuracy did not differ among age groups.

In addition, the fMRI task was designed to be well within a child's ability so that accuracy would be very high. While this design was effective with regard to compliance (no child had to be excluded), it resulted in a restricted range with regard to in‐scanner task accuracy, which as a result, might have limited the ability to detect a correlation.

A further drawback of our study is the slice thickness of 4 mm with a 1‐mm gap. While a thickness of 4 mm or more is common in studies evaluating language lateralization in children (Berl, Mayo, et al., 2014; Berl, Zimmaro, et al., 2014; Elkana, Frost, Kramer, Ben‐Bashat, & Schweiger, 2013; Everts et al., 2010; Sepeta et al., 2016; Szaflarski et al., 2014; Westmacott, McAndrews, & deVeber, 2017) as it allows shorter acquisition times when covering the whole brain, thinner slices would possibly have further enhanced the detection of the BOLD signal.

The relatively small sample size of 35 children and adolescents, taking into account the large age range of participants, is a further limitation of our study. A possible influence of age on language lateralization may become statistically significant investigating a larger study sample as the strength of age is supposed to have a significant but relatively small effect on lateralization: Berl, Mayo, et al. (2014) have shown that age only accounts for 5% of variance in change in LI over age. In addition, in‐scanner performance data are missing from 11 participants, thus further reducing the sample size with regard to this measurement. Furthermore, we investigated only right‐handed children. Future studies may therefore account for the association of left‐handedness with language abilities and their relationship with hemispheric lateralization.

### 4.8 Conclusions

The results of the present study indicate that better verbal abilities in and out of the scanner go along with less lateralization of semantic language activation. Less lateralization in the overall brain was associated with better in‐scanner task accuracy on a semantic language decision task and out‐of‐scanner vocabulary and verbal fluency. Specifically, different regions were correlated with different aspects of verbal performance. Less lateralization in the frontal lobes was associated with better in‐scanner semantic language decision and out‐of‐scanner verbal fluency, and less lateralization in the parietal lobes was associated with better out‐of‐scanner verbal learning. On the contrary, no significant association of language lateralization in the temporal lobes with cognitive performance was observed.
