# Supplementary material for: Weaker semantic language lateralization associated with better semantic language performance in healthy right‐handed children
Source: Brain Behav. 2018 Oct 8;8(11):e01072. doi: 10.1002/brb3.1072 (PMC6236252; doi:10.1002/brb3.1072)
Supplement: Supplementary file 1 [file BRB3-8-e01072-s001.docx]

**Supplementary information to fMRI age-groups**

For the fMRI paradigm, three different age-adjusted versions were available (7–9 years old, 10–12 years old, 13-16 years old). The Supplement table shows demographic data, framewise displacement, and performance data splitted in the three fMRI age-groups.

Statistical difference by fMRI age-groups was calculated using Kruskal-Wallis H-Test. Age-groups did not differ statistically in handedness or sex. Furthermore, head movement during fMRI measurement, mean correct responses during the in-scanner language task, and mean in-scanner reaction times were not significantly different between groups. Thus, the three fMRI age-groups were comparable with regard to sex, handedness, in-scanner movement, in-scanner task performance, and in-scanner reaction time.

Supplement table. Demographic and in-scanner data by fMRI age-groups

|  |  |  |  |  |
| --- | --- | --- | --- | --- |
|  | Age group 1 | Age group 2 | Age group 3 | Difference by groups |
| n | 16 | 11 | 8 |  |
| sex (f/m) | 8/8 | 3/8 | 3/5 | .499 |
| handedness mean, SD (range) | 95.63, 9.64 (70-100) | 97.27, 4.67  (90-100) | 92.50, 17.53 (50-100) | .947 |
|  |  |  |  |  |
| framewise displacement |  |  |  |  |
| mean of mean movement in mm, SD (range) | .08, .03  (.04-.13) | .08, .04  (.03-.15) | .08, .05  (.03-.18) | .938 |
|  |  |  |  |  |
| in-scanner language task |  |  |  |  |
| n | 13 | 7 | 4 |  |
| task performance in percent correct mean, SD (range) | 91.23, 3.93 (80-100) | 96.29, 3.76  (80-100) | 98.00, 1.40 (894-100) | .129 |
| reaction time in ms mean, SD (range) | 3034, 320  (2339-3739) | 3093, 245 (2732-3440) | 2819, 301 (2391-3369) | .274 |

Note: In-scanner task performance and reaction times were missing due to technical reasons in 11 participants.”
